# Are rich club regions masters or slaves of brain network dynamics?

**DOI:** 10.1186/1471-2202-16-S1-P18

**Published:** 2015-12-18

**Authors:** Leonardo L Gollo, Andrew Zalesky, R Matthew Hutchison, Martijn van den Heuvel, Michael Breakspear

**Affiliations:** 1 Systems Neuroscience Group, QIMR Berghofer, Brisbane, Queensland, QLD 4006, Australia; 2 Melbourne Neuropsychiatry Centre, Melbourne, Victoria, Australia; 3 Melbourne Health, Melbourne, Victoria, Australia; 4 Center for Brain Science, Harvard University, Cambridge, MA, USA; 5 Brain Center Rudolf Magnus, University Medical Center, Utrecht, the Netherlands

## 

Anatomical networks of brain systems emphasize their inherent hierarchical structure, in which the rich club (most inner core of the brain) is highlighted because it promotes topological efficiency and integrates communication between regions [1]. The hierarchical structure is fundamental and also observed in the neuronal dynamics: Cortical anatomy is thought to recapitulate the temporal hierarchy that is inherent in the dynamics of environmental states and human behavior [2]. According to this classical view the caudal-rostral arrangement maps the fast-slow gradient of neuronal time scales [3]. Here we ask whether the topological arrangement of the networks can provide a scaffold on which a hierarchy of time scales including slow and stable dynamics emerges. We modeled neuronal dynamics unfolding on an anatomical network reconstructed from primate cortex that has a set of rich-club regions at the core of the network that are surrounded by their feeders and peripheral areas [4]. We utilized a conductance-based neural mass model [5], which gives rise to node dynamics with two time scales. The system synchronizes at the fast time scale and exhibits rich phase relations at the slow rhythm (~10Hz). We then characterized and compared the oscillatory dynamics of nodes and the synchronization between pairs of nodes. Our results show that highly connected regions, such as rich-club members, exhibit a more regular dynamics, and larger stability of phase relation with respective to other regions [6]. Owing to the dynamics of the local connectivity and network-motif structure [7,8], central nodes also catalyze better local zero-lag synchronization between their neighbors. Together our results show how the topology of this constellation of brain regions supports stable, slowly fluctuating patterns of synchronization. In contrast, the topology of the surrounding "feeder" cortical regions shows unstable, rapidly fluctuating dynamics. Hence, assuming the interaction of identical dynamical elements coupled via the structural connectome of primate cortex [4], we already find an emerging hierarchy of time scales due to the network structure. We thus propose that the network properties of central cortical regions are the structural determinants of slow neuronal fluctuations in these brain regions. Although the traditional caudal-rostral hierarchical cortical mapping exhibited an unprecedented success, our dynamic-based study aligned with recent observations indicates that the cortical organizing principle may be best described by a periphery-core axis. Intriguingly, as a metaphor to social networks, rich-club members could be seen to be "slaves" of their own power. The richness of these brain regions comes from their large in-degree. However, for this very reason, their dynamics are consequently more regular because larger in-degree guarantees larger regularity. In other words, the autonomous dynamics of connectome hubs are largely enslaved to the strong, rhythmic output of the entire connectome. Therefore, rich-club regions cannot behave very differently from what is "expected" in contrast to the peripheral nodes, which have the most freedom to explore the dynamical landscape.

## References

[B1] van den Heuvel MP Sporns O Network hubs in the human brain Trends Cogn Sci 2013 17 12 683 696 24231140 10.1016/j.tics.2013.09.012

[B2] Kiebel SJ Daunizeau J Friston KJ A hierarchy of time-scales and the brain PLoS CB 2008 4 11 e1000209 10.1371/journal.pcbi.1000209 PMC2568860 19008936

[B3] Fuster JM The prefrontal cortex-an update: time is of the essence Neuron 2001 30 2 319 333 11394996 10.1016/s0896-6273(01)00285-9

[B4] Harriger L Van Den Heuvel MP Sporns O Rich club organization of macaque cerebral cortex and its role in network communication PloS One 2012 7 9 e46497 23029538 10.1371/journal.pone.0046497 PMC3460908

[B5] Breakspear M Terry JR Friston KJ Modulation of excitatory synaptic coupling facilitates synchronization and complex dynamics in a biophysical model of neuronal dynamics Network 2003 14 4 703 732 14653499

[B6] Gollo LL Zalesky A Hutchison RM van den Heuvel M Breakspear M Dwelling quietly in the rich club: brain network determinants of slow cortical fluctuations Phil Trans R Soc B 2015 370 1668 10.1098/rstb.2014.0165 PMC4387508 25823864

[B7] Gollo LL Mirasso C Sporns O Breakspear M Mechanisms of zero-lag synchronization in cortical motifs PLoS Comput Biol 2014 10 e1003548 24763382 10.1371/journal.pcbi.1003548 PMC3998884

[B8] Gollo LL Breakspear M The frustrated brain: from dynamics on motifs to communities and networks Phil Trans R Soc B 2014 369 1653 20130532 25180310 10.1098/rstb.2013.0532 PMC4150307

